# Supplementary material for: Diffusion MRS tracks distinct trajectories of neuronal development in the cerebellum and thalamus of rat neonates
Source: eLife. 2025 Oct 9;13:RP96625. doi: 10.7554/eLife.96625 (PMC12510685; doi:10.7554/eLife.96625)
Supplement: Supplementary file 1. [file elife-96625-supp1.docx]

| *HIGH B VALUES* | | | |  | | |  | | |  | | |  | | |  | | |  | | |  | | |  | | |  | | |  | |
| --- | --- | --- | --- | --- | --- | --- | --- | --- | --- | --- | --- | --- | --- | --- | --- | --- | --- | --- | --- | --- | --- | --- | --- | --- | --- | --- | --- | --- | --- | --- | --- | --- |
| **CEREBELLUM** | |  | |  | | |  | | |  | | |  | | |  | | |  | | |  | | |  | | |  | | |  | |
| tNAA |  | |  | | |  | | |  | | |  | | |  | | Glu | | |  | | |  | | |  | | |  | | |  |
| b-value | P5 | | P10 | | | P15 | | | P20 | | | P30 | | |  | | b-value | | | P5 | | | P10 | | | P15 | | | P20 | | | P30 |
| 0.035 | 8.17 | | 6.67 | | | 2.67 | | | 2.00 | | | 2.25 | | |  | | 0.035 | | | 8.00 | | | 6.22 | | | 3.00 | | | 2.63 | | | 3.25 |
| 3.035 | 11.33 | | 7.67 | | | 3.11 | | | 2.38 | | | 2.88 | | |  | | 3.035 | | | 10.00 | | | 7.11 | | | 3.33 | | | 3.00 | | | 3.88 |
| 6 | 10.83 | | 7.56 | | | 3.44 | | | 2.50 | | | 3.00 | | |  | | 6 | | | 11.17 | | | 7.44 | | | 3.44 | | | 3.13 | | | 4.13 |
| 10 | 12.40 | | 9.33 | | | 4.00 | | | 3.00 | | | 3.38 | | |  | | 10 | | | 14.40 | | | 8.33 | | | 4.00 | | | 3.38 | | | 4.38 |
| 20 | 22.50 | | 11.78 | | | 4.56 | | | 3.13 | | | 4.00 | | |  | | 20 | | | 21.75 | | | 11.56 | | | 4.56 | | | 4.00 | | | 5.50 |
| 30 | 31.00 | | 14.11 | | | 5.78 | | | 4.25 | | | 5.75 | | |  | | 30 | | | 33.75 | | | 15.00 | | | 5.89 | | | 5.00 | | | 7.63 |
|  |  | |  | | |  | | |  | | |  | | |  | |  | | |  | | |  | | |  | | |  | | |  |
|  |  | |  | | |  | | |  | | |  | | |  | |  | | |  | | |  | | |  | | |  | | |  |
| tCr |  | |  | | |  | | |  | | |  | | |  | | Tau | | |  | | |  | | |  | | |  | | |  |
| b-value | P5 | | P10 | | | P15 | | | P20 | | | P30 | | |  | | b-value | | | P5 | | | P10 | | | P15 | | | P20 | | | P30 |
| 0.035 | 4.00 | | 2.89 | | | 1.22 | | | 1.25 | | | 2.00 | | |  | | 0.035 | | | 3.17 | | | 3.67 | | | 2.11 | | | 2.25 | | | 3.38 |
| 3.035 | 5.50 | | 3.22 | | | 2.00 | | | 1.88 | | | 2.00 | | |  | | 3.035 | | | 4.17 | | | 4.22 | | | 2.56 | | | 2.75 | | | 4.13 |
| 6 | 5.17 | | 3.33 | | | 2.11 | | | 1.88 | | | 2.13 | | |  | | 6 | | | 4.33 | | | 4.44 | | | 2.67 | | | 2.75 | | | 4.00 |
| 10 | 6.40 | | 3.78 | | | 2.22 | | | 2.00 | | | 2.38 | | |  | | 10 | | | 5.00 | | | 5.11 | | | 3.33 | | | 3.13 | | | 5.00 |
| 20 | 9.75 | | 5.22 | | | 2.22 | | | 2.13 | | | 3.00 | | |  | | 20 | | | 8.00 | | | 6.56 | | | 3.44 | | | 3.63 | | | 5.88 |
| 30 | 18.00 | | 7.22 | | | 3.33 | | | 2.75 | | | 4.13 | | |  | | 30 | | | 27.00 | | | 8.67 | | | 4.33 | | | 4.50 | | | 8.75 |
|  |  | |  | | |  | | |  | | |  | | |  | |  | | |  | | |  | | |  | | |  | | |  |
|  |  | |  | | |  | | |  | | |  | | |  | |  | | |  | | |  | | |  | | |  | | |  |
| tCho |  | |  | | |  | | |  | | |  | | |  | | Ins | | |  | | |  | | |  | | |  | | |  |
| b-value | P5 | | P10 | | | P15 | | | P20 | | | P30 | | |  | | b-value | | | P5 | | | P10 | | | P15 | | | P20 | | | P30 |
| 0.035 | 5.83 | | 7.00 | | | 3.67 | | | 4.50 | | | 5.00 | | |  | | 0.035 | | | 10.50 | | | 15.56 | | | 7.00 | | | 6.00 | | | 6.38 |
| 3.035 | 7.50 | | 6.11 | | | 3.89 | | | 4.75 | | | 5.63 | | |  | | 3.035 | | | 12.83 | | | 18.11 | | | 7.56 | | | 7.00 | | | 7.00 |
| 6 | 7.33 | | 6.00 | | | 4.67 | | | 4.88 | | | 5.38 | | |  | | 6 | | | 20.50 | | | 20.89 | | | 9.44 | | | 7.00 | | | 7.25 |
| 10 | 8.80 | | 6.89 | | | 5.33 | | | 5.13 | | | 6.63 | | |  | | 10 | | | 22.40 | | | 137.56 | | | 12.00 | | | 8.63 | | | 8.50 |
| 20 | 9.75 | | 8.67 | | | 6.33 | | | 6.50 | | | 8.00 | | |  | | 20 | | | 267.75 | | | 309.44 | | | 17.33 | | | 13.50 | | | 12.25 |
| 30 | 20.50 | | 14.33 | | | 8.44 | | | 8.25 | | | 12.38 | | |  | | 30 | | | 533.50 | | | 505.89 | | | 133.78 | | | 32.75 | | | 21.75 |
|  |  | |  | | |  | | |  | | |  | | |  | |  | | |  | | |  | | |  | | |  | | |  |
|  |  | |  | | |  | | |  | | |  | | |  | |  | | |  | | |  | | |  | | |  | | |  |
| **THALAMUS** | |  | |  | | |  | | |  | | |  | | |  | | |  | | |  | | |  | | |  | | |  | |
| NAA |  | |  | | |  | | |  | | |  | | |  | | Glu | | |  | | |  | | |  | | |  | | |  |
| b-value | P5 | | P10 | | | P15 | | | P20 | | | P30 | | |  | | b-value | | | P5 | | | P10 | | | P15 | | | P20 | | | P30 |
| 0.035 | 6.38 | | 3.00 | | | 2.00 | | | 2.00 | | | 2.00 | | |  | | 0.035 | | | 5.88 | | | 3.89 | | | 3.00 | | | 3.00 | | | 3.11 |
| 3.035 | 7.88 | | 4.11 | | | 2.22 | | | 2.11 | | | 2.67 | | |  | | 3.035 | | | 7.88 | | | 5.44 | | | 3.56 | | | 3.67 | | | 4.33 |
| 6 | 7.38 | | 4.22 | | | 2.22 | | | 2.11 | | | 2.89 | | |  | | 6 | | | 8.38 | | | 6.00 | | | 3.67 | | | 3.89 | | | 4.56 |
| 10 | 9.63 | | 5.11 | | | 3.11 | | | 3.00 | | | 3.56 | | |  | | 10 | | | 10.88 | | | 7.22 | | | 4.33 | | | 4.78 | | | 5.56 |
| 20 | 10.57 | | 6.11 | | | 3.44 | | | 3.22 | | | 4.11 | | |  | | 20 | | | 13.29 | | | 9.67 | | | 5.78 | | | 5.89 | | | 6.89 |
| 30 | 16.86 | | 7.78 | | | 4.22 | | | 4.22 | | | 5.33 | | |  | | 30 | | | 29.71 | | | 13.00 | | | 7.22 | | | 7.00 | | | 8.78 |
|  |  | |  | | |  | | |  | | |  | | |  | |  | | |  | | |  | | |  | | |  | | |  |
|  |  | |  | | |  | | |  | | |  | | |  | |  | | |  | | |  | | |  | | |  | | |  |
| tCr |  | |  | | |  | | |  | | |  | | |  | | Tau | | |  | | |  | | |  | | |  | | |  |
| b-value | P5 | | P10 | | | P15 | | | P20 | | | P30 | | |  | | b-value | | | P5 | | | P10 | | | P15 | | | P20 | | | P30 |
| 0.035 | 3.25 | | 2.00 | | | 2.00 | | | 2.00 | | | 2.33 | | |  | | 0.035 | | | 2.38 | | | 2.00 | | | 2.11 | | | 3.56 | | | 5.00 |
| 3.035 | 4.00 | | 2.89 | | | 2.11 | | | 2.11 | | | 2.89 | | |  | | 3.035 | | | 3.13 | | | 2.89 | | | 3.00 | | | 4.44 | | | 6.56 |
| 6 | 4.25 | | 3.00 | | | 2.11 | | | 2.44 | | | 3.11 | | |  | | 6 | | | 3.25 | | | 3.00 | | | 3.11 | | | 4.67 | | | 7.67 |
| 10 | 5.50 | | 3.78 | | | 3.11 | | | 3.22 | | | 3.67 | | |  | | 10 | | | 4.13 | | | 3.78 | | | 3.78 | | | 5.78 | | | 8.33 |
| 20 | 6.29 | | 4.78 | | | 3.56 | | | 4.33 | | | 4.78 | | |  | | 20 | | | 4.71 | | | 4.78 | | | 4.67 | | | 7.33 | | | 10.44 |
| 30 | 10.57 | | 6.33 | | | 4.56 | | | 5.33 | | | 6.56 | | |  | | 30 | | | 8.14 | | | 5.89 | | | 5.89 | | | 8.78 | | | 13.11 |
|  |  | |  | | |  | | |  | | |  | | |  | |  | | |  | | |  | | |  | | |  | | |  |
|  |  | |  | | |  | | |  | | |  | | |  | |  | | |  | | |  | | |  | | |  | | |  |
| tCho |  | |  | | |  | | |  | | |  | | |  | | Ins | | |  | | |  | | |  | | |  | | |  |
| b-value | P5 | | P10 | | | P15 | | | P20 | | | P30 | | |  | | b-value | | | P5 | | | P10 | | | P15 | | | P20 | | | P30 |
| 0.035 | 5.75 | | 3.78 | | | 3.89 | | | 4.00 | | | 5.22 | | |  | | 0.035 | | | 10.88 | | | 13.11 | | | 15.67 | | | 9.56 | | | 6.67 |
| 3.035 | 6.88 | | 5.11 | | | 4.33 | | | 4.89 | | | 5.56 | | |  | | 3.035 | | | 12.63 | | | 14.00 | | | 23.44 | | | 11.56 | | | 8.56 |
| 6 | 6.38 | | 4.89 | | | 4.44 | | | 4.78 | | | 6.89 | | |  | | 6 | | | 19.13 | | | 19.78 | | | 28.00 | | | 12.33 | | | 9.56 |
| 10 | 8.00 | | 6.11 | | | 5.11 | | | 6.00 | | | 6.67 | | |  | | 10 | | | 165.50 | | | 135.11 | | | 158.67 | | | 15.56 | | | 15.33 |
| 20 | 8.43 | | 7.22 | | | 5.67 | | | 6.33 | | | 7.67 | | |  | | 20 | | | 339.14 | | | 607.78 | | | 863.33 | | | 248.00 | | | 38.33 |
| 30 | 12.14 | | 8.89 | | | 6.44 | | | 7.22 | | | 9.56 | | |  | | 30 | | | 513.00 | | | 483.89 | | | 908.00 | | | 583.11 | | | 47.00 |
|  |  | |  | | |  | | |  | | |  | | |  | |  | | |  | | |  | | |  | | |  | | |  |
| *LONG DIFFUSION TIMES* | | | | |  | | |  | | |  | | |  | | | |  | | |  | | |  | | |  | | |  | | |
| **CEREBELLUM** | |  | |  | | |  | | |  | | |  | | |  | | |  | | |  | | |  | | |  | | |  | |
| tNAA |  | |  | | |  | | |  | | |  | | |  | | Glu | | |  | | |  | | |  | | |  | | |  |
| TM (b0) | P5 | | P10 | | | P15 | | | P20 | | | P30 | | |  | | TM (b0) | | | P5 | | | P10 | | | P15 | | | P20 | | | P30 |
| 100 | 8.17 | | 6.13 | | | 3.00 | | | 2.43 | | | 2.17 | | |  | | 100 | | | 8.17 | | | 5.75 | | | 3.22 | | | 5.29 | | | 3.17 |
| 500 | 8.25 | | 5.94 | | | 2.67 | | | 2.36 | | | 2.00 | | |  | | 500 | | | 7.83 | | | 5.69 | | | 3.22 | | | 4.79 | | | 3.25 |
| 750 | 8.00 | | 5.88 | | | 2.67 | | | 2.23 | | | 2.00 | | |  | | 750 | | | 7.88 | | | 5.71 | | | 3.26 | | | 4.05 | | | 3.28 |
| 1000 | 8.57 | | 6.16 | | | 2.75 | | | 2.53 | | | 2.04 | | |  | | 1000 | | | 7.96 | | | 5.94 | | | 3.31 | | | 4.57 | | | 3.50 |
| TM (b0+3) | P5 | | P10 | | | P15 | | | P20 | | | P30 | | |  | | TM (b0+3) | | | P5 | | | P10 | | | P15 | | | P20 | | | P30 |
| 100 | 11.67 | | 8.00 | | | 3.33 | | | 3.14 | | | 3.17 | | |  | | 100 | | | 9.33 | | | 7.25 | | | 3.22 | | | 5.86 | | | 4.00 |
| 500 | 10.92 | | 7.31 | | | 3.17 | | | 2.79 | | | 2.67 | | |  | | 500 | | | 8.92 | | | 6.81 | | | 3.17 | | | 5.14 | | | 3.92 |
| 750 | 10.44 | | 7.00 | | | 3.19 | | | 2.50 | | | 2.50 | | |  | | 750 | | | 9.19 | | | 6.79 | | | 3.26 | | | 4.32 | | | 4.00 |
| 1000 | 11.04 | | 7.26 | | | 3.25 | | | 2.73 | | | 2.54 | | |  | | 1000 | | | 9.65 | | | 6.87 | | | 3.33 | | | 5.23 | | | 4.08 |
|  |  | |  | | |  | | |  | | |  | | |  | |  | | |  | | |  | | |  | | |  | | |  |
|  |  | |  | | |  | | |  | | |  | | |  | |  | | |  | | |  | | |  | | |  | | |  |
| tCr |  | |  | | |  | | |  | | |  | | |  | | Tau | | |  | | |  | | |  | | |  | | |  |
| TM (b0) | P5 | | P10 | | | P15 | | | P20 | | | P30 | | |  | | TM (b0) | | | P5 | | | P10 | | | P15 | | | P20 | | | P30 |
| 100 | 3.83 | | 2.75 | | | 1.44 | | | 1.43 | | | 1.67 | | |  | | 100 | | | 3.17 | | | 3.25 | | | 2.33 | | | 2.14 | | | 3.00 |
| 500 | 3.75 | | 2.56 | | | 1.33 | | | 1.43 | | | 1.75 | | |  | | 500 | | | 2.83 | | | 3.06 | | | 2.22 | | | 2.14 | | | 3.00 |
| 750 | 3.69 | | 2.54 | | | 1.41 | | | 1.41 | | | 1.72 | | |  | | 750 | | | 2.75 | | | 2.88 | | | 2.22 | | | 2.14 | | | 2.89 |
| 1000 | 3.96 | | 2.74 | | | 1.58 | | | 1.53 | | | 1.79 | | |  | | 1000 | | | 2.74 | | | 2.94 | | | 2.28 | | | 2.13 | | | 2.92 |
| TM (b0+3) | P5 | | P10 | | | P15 | | | P20 | | | P30 | | |  | | TM (b0+3) | | | P5 | | | P10 | | | P15 | | | P20 | | | P30 |
| 100 | 4.83 | | 3.25 | | | 2.00 | | | 1.71 | | | 2.17 | | |  | | 100 | | | 4.00 | | | 4.13 | | | 2.67 | | | 2.57 | | | 4.50 |
| 500 | 4.58 | | 3.06 | | | 1.83 | | | 1.71 | | | 2.08 | | |  | | 500 | | | 3.50 | | | 3.88 | | | 2.44 | | | 2.50 | | | 3.83 |
| 750 | 4.44 | | 3.08 | | | 1.85 | | | 1.64 | | | 2.06 | | |  | | 750 | | | 3.31 | | | 3.58 | | | 2.41 | | | 2.41 | | | 3.72 |
| 1000 | 4.74 | | 3.16 | | | 1.97 | | | 1.70 | | | 2.13 | | |  | | 1000 | | | 3.30 | | | 3.48 | | | 2.42 | | | 2.43 | | | 3.79 |
|  |  | |  | | |  | | |  | | |  | | |  | |  | | |  | | |  | | |  | | |  | | |  |
|  |  | |  | | |  | | |  | | |  | | |  | |  | | |  | | |  | | |  | | |  | | |  |
|  |  | |  | | |  | | |  | | |  | | |  | |  | | |  | | |  | | |  | | |  | | |  |
| tCho |  | |  | | |  | | |  | | |  | | |  | | Ins | | |  | | |  | | |  | | |  | | |  |
| TM (b0) | P5 | | P10 | | | P15 | | | P20 | | | P30 | | |  | | TM (b0) | | | P5 | | | P10 | | | P15 | | | P20 | | | P30 |
| 100 | 5.50 | | 5.38 | | | 4.00 | | | 4.14 | | | 4.33 | | |  | | 100 | | | 12.17 | | | 15.50 | | | 7.89 | | | 7.71 | | | 5.50 |
| 500 | 5.58 | | 5.06 | | | 3.78 | | | 4.14 | | | 4.50 | | |  | | 500 | | | 10.00 | | | 13.44 | | | 7.28 | | | 6.93 | | | 5.50 |
| 750 | 5.50 | | 4.92 | | | 3.93 | | | 4.09 | | | 4.61 | | |  | | 750 | | | 9.75 | | | 13.42 | | | 8.11 | | | 6.27 | | | 5.39 |
| 1000 | 5.78 | | 5.06 | | | 4.50 | | | 4.23 | | | 4.75 | | |  | | 1000 | | | 9.83 | | | 14.10 | | | 8.72 | | | 6.53 | | | 5.46 |
| TM (b0+3) | P5 | | P10 | | | P15 | | | P20 | | | P30 | | |  | | TM (b0+3) | | | P5 | | | P10 | | | P15 | | | P20 | | | P30 |
| 100 | 7.33 | | 5.63 | | | 4.22 | | | 5.00 | | | 6.67 | | |  | | 100 | | | 12.83 | | | 17.63 | | | 8.00 | | | 8.00 | | | 7.33 |
| 500 | 6.58 | | 5.44 | | | 4.06 | | | 4.64 | | | 6.00 | | |  | | 500 | | | 11.08 | | | 15.50 | | | 7.28 | | | 7.36 | | | 6.58 |
| 750 | 6.56 | | 5.33 | | | 4.11 | | | 4.50 | | | 5.89 | | |  | | 750 | | | 10.56 | | | 14.33 | | | 7.44 | | | 6.73 | | | 6.22 |
| 1000 | 6.74 | | 5.29 | | | 4.31 | | | 4.57 | | | 5.96 | | |  | | 1000 | | | 10.87 | | | 14.90 | | | 8.36 | | | 6.83 | | | 6.38 |
|  |  | |  | | |  | | |  | | |  | | |  | |  | | |  | | |  | | |  | | |  | | |  |
|  |  | |  | | |  | | |  | | |  | | |  | |  | | |  | | |  | | |  | | |  | | |  |
|  |  | |  | | |  | | |  | | |  | | |  | |  | | |  | | |  | | |  | | |  | | |  |
| **THALAMUS** | |  | |  | | |  | | |  | | |  | | |  | | |  | | |  | | |  | | |  | | |  | |
| tNAA |  | |  | | |  | | |  | | |  | | |  | | Glu | | |  | | |  | | |  | | |  | | |  |
| TM (b0) | P5 | | P10 | | | P15 | | | P20 | | | P30 | | |  | | TM (b0) | | | P5 | | | P10 | | | P15 | | | P20 | | | P30 |
| 100 | 6.63 | | 3.22 | | | 2.11 | | | 2.00 | | | 2.00 | | |  | | 100 | | | 6.13 | | | 4.00 | | | 3.11 | | | 3.00 | | | 3.33 |
| 500 | 7.07 | | 3.11 | | | 2.06 | | | 2.00 | | | 2.00 | | |  | | 500 | | | 6.27 | | | 4.06 | | | 3.06 | | | 3.06 | | | 3.29 |
| 750 | 7.87 | | 3.15 | | | 2.07 | | | 2.00 | | | 2.00 | | |  | | 750 | | | 6.48 | | | 4.11 | | | 3.07 | | | 3.11 | | | 3.35 |
| 1000 | 8.55 | | 3.28 | | | 2.08 | | | 2.00 | | | 2.00 | | |  | | 1000 | | | 6.81 | | | 4.31 | | | 3.08 | | | 3.22 | | | 3.49 |
| TM (b0+3) | P5 | | P10 | | | P15 | | | P20 | | | P30 | | |  | | TM (b0+3) | | | P5 | | | P10 | | | P15 | | | P20 | | | P30 |
| 100 | 8.38 | | 4.22 | | | 2.33 | | | 2.56 | | | 2.78 | | |  | | 100 | | | 7.75 | | | 5.56 | | | 3.67 | | | 4.11 | | | 4.33 |
| 500 | 8.07 | | 4.00 | | | 2.17 | | | 2.28 | | | 2.59 | | |  | | 500 | | | 7.67 | | | 5.44 | | | 3.39 | | | 4.06 | | | 4.24 |
| 750 | 8.00 | | 4.04 | | | 2.15 | | | 2.19 | | | 2.50 | | |  | | 750 | | | 7.70 | | | 5.52 | | | 3.44 | | | 4.00 | | | 4.35 |
| 1000 | 8.55 | | 4.06 | | | 2.14 | | | 2.17 | | | 2.51 | | |  | | 1000 | | | 7.97 | | | 5.64 | | | 3.53 | | | 4.06 | | | 4.51 |
|  |  | |  | | |  | | |  | | |  | | |  | |  | | |  | | |  | | |  | | |  | | |  |
|  |  | |  | | |  | | |  | | |  | | |  | |  | | |  | | |  | | |  | | |  | | |  |
| tCr |  | |  | | |  | | |  | | |  | | |  | | Tau | | |  | | |  | | |  | | |  | | |  |
| TM (b0) | P5 | | P10 | | | P15 | | | P20 | | | P30 | | |  | | TM (b0) | | | P5 | | | P10 | | | P15 | | | P20 | | | P30 |
| 100 | 3.50 | | 2.11 | | | 2.00 | | | 2.11 | | | 2.33 | | |  | | 100 | | | 2.38 | | | 2.00 | | | 2.11 | | | 3.67 | | | 5.00 |
| 500 | 3.47 | | 2.11 | | | 2.00 | | | 2.06 | | | 2.29 | | |  | | 500 | | | 2.47 | | | 2.00 | | | 2.06 | | | 3.56 | | | 5.00 |
| 750 | 3.57 | | 2.15 | | | 2.04 | | | 2.04 | | | 2.35 | | |  | | 750 | | | 2.48 | | | 2.00 | | | 2.07 | | | 3.44 | | | 4.96 |
| 1000 | 3.74 | | 2.31 | | | 2.08 | | | 2.08 | | | 2.49 | | |  | | 1000 | | | 2.61 | | | 2.06 | | | 2.11 | | | 3.47 | | | 5.17 |
| TM (b0+3) | P5 | | P10 | | | P15 | | | P20 | | | P30 | | |  | | TM (b0+3) | | | P5 | | | P10 | | | P15 | | | P20 | | | P30 |
| 100 | 4.00 | | 2.78 | | | 2.11 | | | 2.89 | | | 2.89 | | |  | | 100 | | | 3.13 | | | 2.89 | | | 3.00 | | | 5.78 | | | 6.67 |
| 500 | 3.93 | | 2.61 | | | 2.06 | | | 2.50 | | | 2.88 | | |  | | 500 | | | 3.00 | | | 2.56 | | | 2.78 | | | 4.94 | | | 6.47 |
| 750 | 3.96 | | 2.70 | | | 2.07 | | | 2.44 | | | 2.88 | | |  | | 750 | | | 2.91 | | | 2.41 | | | 2.74 | | | 4.63 | | | 6.27 |
| 1000 | 4.16 | | 2.78 | | | 2.14 | | | 2.56 | | | 3.03 | | |  | | 1000 | | | 2.94 | | | 2.36 | | | 2.72 | | | 4.58 | | | 6.43 |
|  |  | |  | | |  | | |  | | |  | | |  | |  | | |  | | |  | | |  | | |  | | |  |
|  |  | |  | | |  | | |  | | |  | | |  | |  | | |  | | |  | | |  | | |  | | |  |
|  |  | |  | | |  | | |  | | |  | | |  | |  | | |  | | |  | | |  | | |  | | |  |
| tCho |  | |  | | |  | | |  | | |  | | |  | | Ins | | |  | | |  | | |  | | |  | | |  |
| TM (b0) | P5 | | P10 | | | P15 | | | P20 | | | P30 | | |  | | TM (b0) | | | P5 | | | P10 | | | P15 | | | P20 | | | P30 |
| 100 | 5.88 | | 4.11 | | | 4.11 | | | 4.44 | | | 5.44 | | |  | | 100 | | | 11.50 | | | 13.33 | | | 16.33 | | | 10.11 | | | 7.44 |
| 500 | 6.40 | | 4.06 | | | 3.94 | | | 4.56 | | | 5.29 | | |  | | 500 | | | 11.20 | | | 13.11 | | | 14.44 | | | 9.28 | | | 6.82 |
| 750 | 92.87 | | 4.30 | | | 4.00 | | | 4.48 | | | 5.12 | | |  | | 750 | | | 11.87 | | | 12.37 | | | 13.15 | | | 8.59 | | | 6.77 |
| 1000 | 135.52 | | 4.58 | | | 4.08 | | | 4.47 | | | 5.14 | | |  | | 1000 | | | 12.39 | | | 12.31 | | | 13.28 | | | 8.58 | | | 6.91 |
| TM (b0+3) | P5 | | P10 | | | P15 | | | P20 | | | P30 | | |  | | TM (b0+3) | | | P5 | | | P10 | | | P15 | | | P20 | | | P30 |
| 100 | 9.00 | | 5.11 | | | 4.67 | | | 6.56 | | | 6.44 | | |  | | 100 | | | 12.38 | | | 16.00 | | | 25.89 | | | 21.89 | | | 9.00 |
| 500 | 7.73 | | 4.94 | | | 4.50 | | | 5.72 | | | 6.00 | | |  | | 500 | | | 11.67 | | | 13.72 | | | 18.39 | | | 15.06 | | | 8.00 |
| 750 | 7.57 | | 5.04 | | | 4.52 | | | 5.37 | | | 5.77 | | |  | | 750 | | | 11.22 | | | 12.96 | | | 15.96 | | | 12.52 | | | 7.65 |
| 1000 | 7.81 | | 5.11 | | | 4.58 | | | 5.42 | | | 5.91 | | |  | | 1000 | | | 10.87 | | | 12.89 | | | 15.44 | | | 11.81 | | | 7.74 |
