# Supplementary material for: Diffusion MRS tracks distinct trajectories of neuronal development in the cerebellum and thalamus of rat neonates
Source: eLife. 2025 Oct 9;13:RP96625. doi: 10.7554/eLife.96625 (PMC12510685; doi:10.7554/eLife.96625)
Supplement: Supplementary file 2. — ‘with WM’ means that the white matter layer was included in the ‘process-like’ layers (in addition to ML). Data come from manual measurements of literature cerebellar figures. References are at the bottom of the Supplementary Information. [file elife-96625-supp2.docx]

| **Nakayama 2018**^1^ | |  |  |  |  |
| --- | --- | --- | --- | --- | --- |
|  | P5 | P8/9 | P13/14 | P20/21 | P60 |
| with WM | 0.65 | 0.68 | 0.53 | 0.4 | 0.37 |
| without WM | 0.88 | 0.79 | 0.61 | 0.46 | 0.4 |
|  |  |  |  |  |  |
|  |  |  |  |  |  |
| **Miterko 2019**^2^ | |  |  |  |  |
|  | P5* | P10 | P15 | P20 | P180 |
| with WM | 0.75 | 0.59 | 0.47 | 0.42 | 0.33 |
| without WM | 0.75 | 0.63 | 0.5 | 0.44 | 0.4 |
|  |  |  |  |  |  |
| *WM not identifiable in the region of lobule IV/V used for the measure | | | | | |
|  |  |  |  |  |  |
|  |  |  |  |  |  |
| **Yamanaka 2004**^3^ | |  |  |  |  |
|  | P5 | P7 | P10 | P15 | P20 |
| without WM | 0.82 | 0.77 | 0.69 | 0.58 | 0.53 |
|  |  |  |  |  |  |
|  |  |  |  |  |  |
| **van der Heijen 2021**^4^ | |  |  |  |  |
|  | P7 |  | P14 |  | Ad |
| without WM | 0.82 |  | 0.53 |  | 0.51 |
|  |  |  |  |  |  |
|  |  |  |  |  |  |
| **Araujo 2019**^5^ |  |  |  |  |  |
|  | P6 | P12 | P15 |  | Ad |
| without WM | 0.87 | 0.76 | 0.64 |  | 0.53 |
|  |  |  |  |  |  |
|  |  |  |  |  |  |
| **Kim and Scott 2014**^6^ | |  |  |  |  |
|  |  |  | P14 |  |  |
| without WM |  |  | 0.43 |  |  |
