# Supplementary material for: Diffusion MRS tracks distinct trajectories of neuronal development in the cerebellum and thalamus of rat neonates
Source: eLife. 2025 Oct 9;13:RP96625. doi: 10.7554/eLife.96625 (PMC12510685; doi:10.7554/eLife.96625)
Supplement: Supplementary file 3. — Supplementary information to Figure 6. [file elife-96625-supp3.docx]

**Supplementary File 3:** Supplementary information to Figure 6**.** Number of 3D neurons reconstructions used from Neuromorph.

Cerebellum P6 - P9 = 4 neurons

Cerebellum P10 = 6 neurons

Cerebellum P11 = 1 neuron

Cerebellum P12 = 3 neurons

Cerebellum P13 = 3 neurons

Cerebellum P35 = 4 neurons

Cerebellum P37 = 1 neuron

Cerebellum P43 = 1 neuron

Thalamus P9 = 1 neuron

Thalamus P10 = 2 neuron

Thalamus P11= 7 neurons

Thalamus P26 = 9 neurons

Thalamus P30 = 2 neurons
